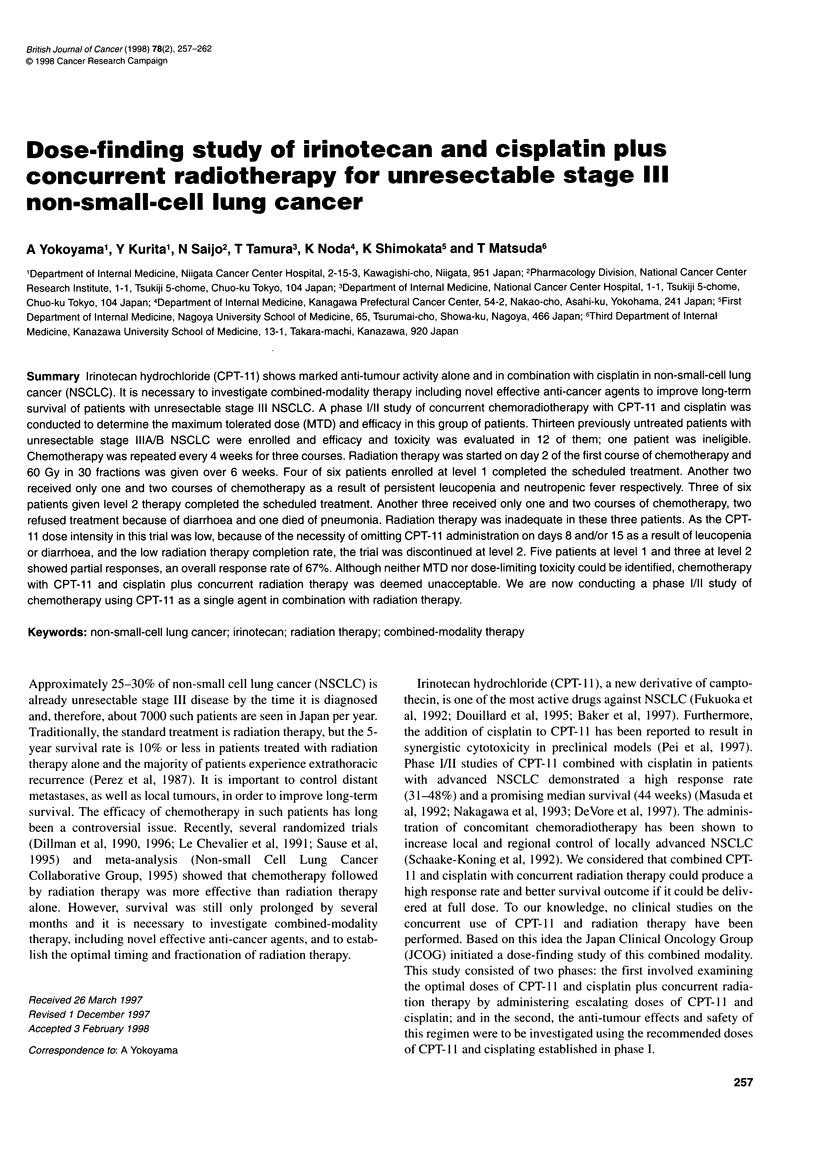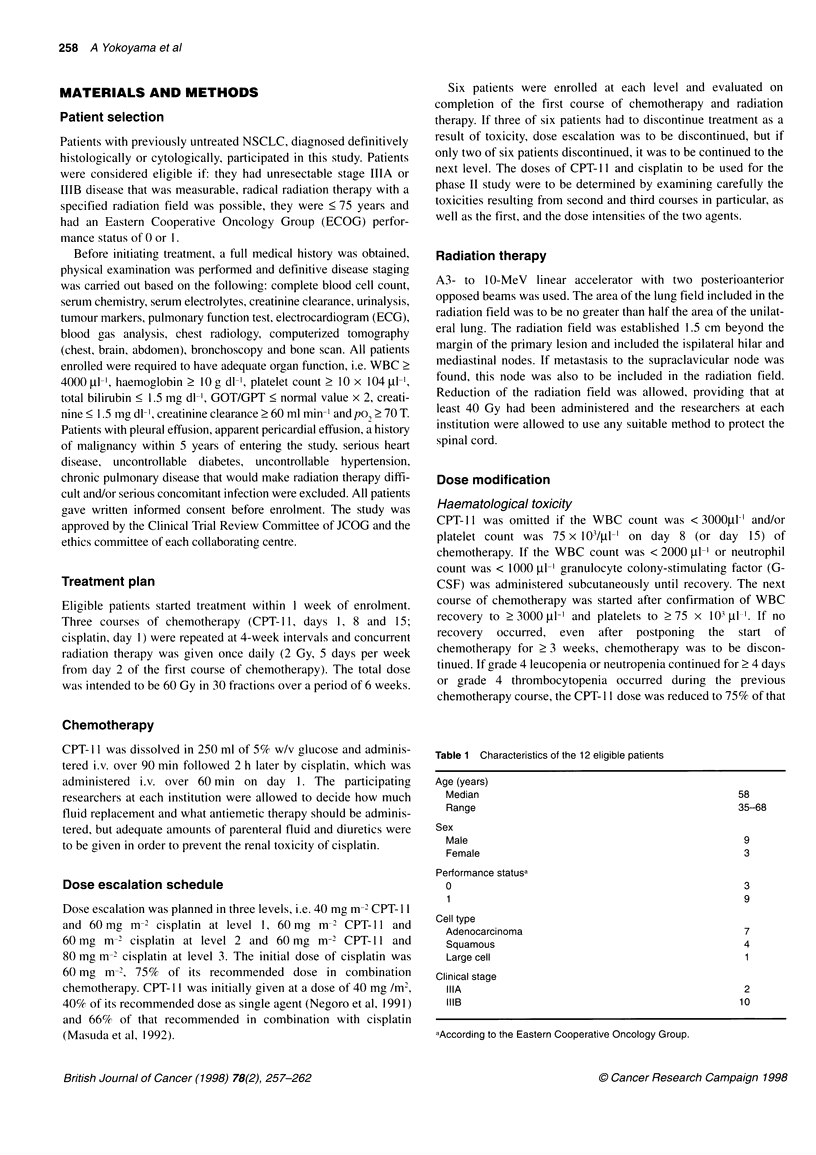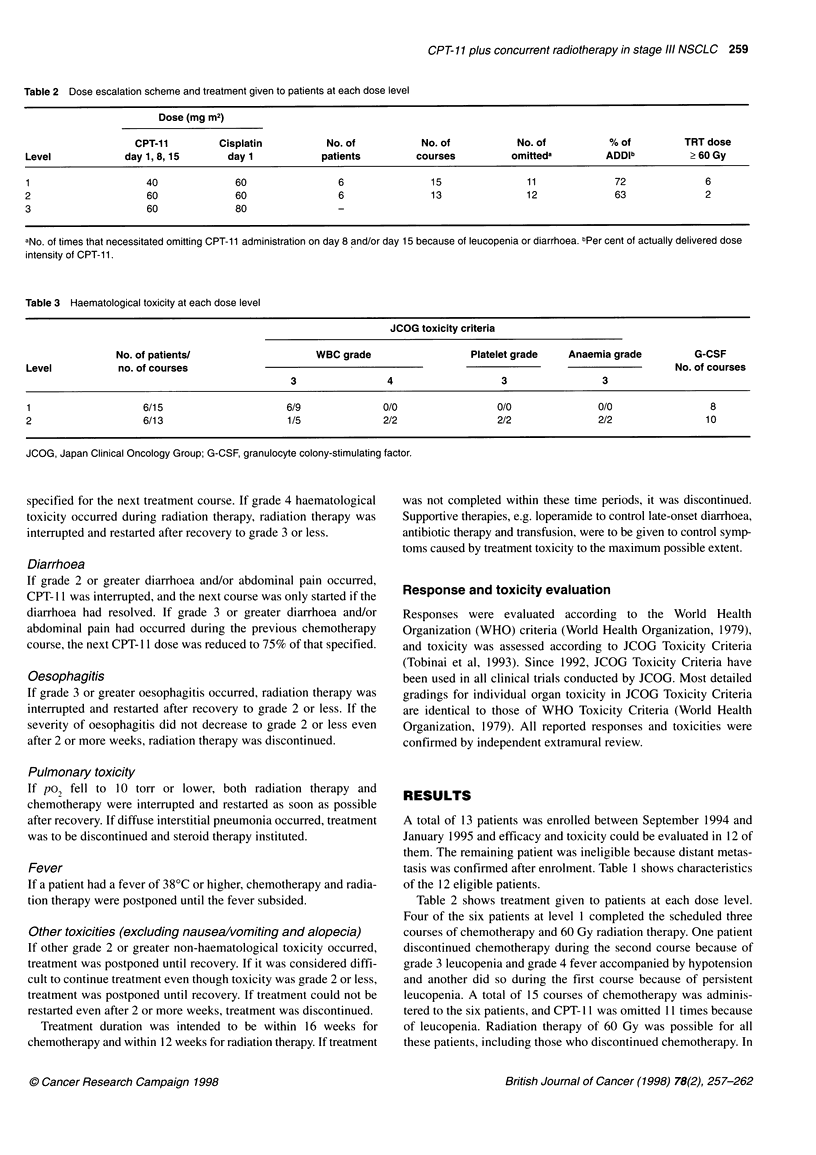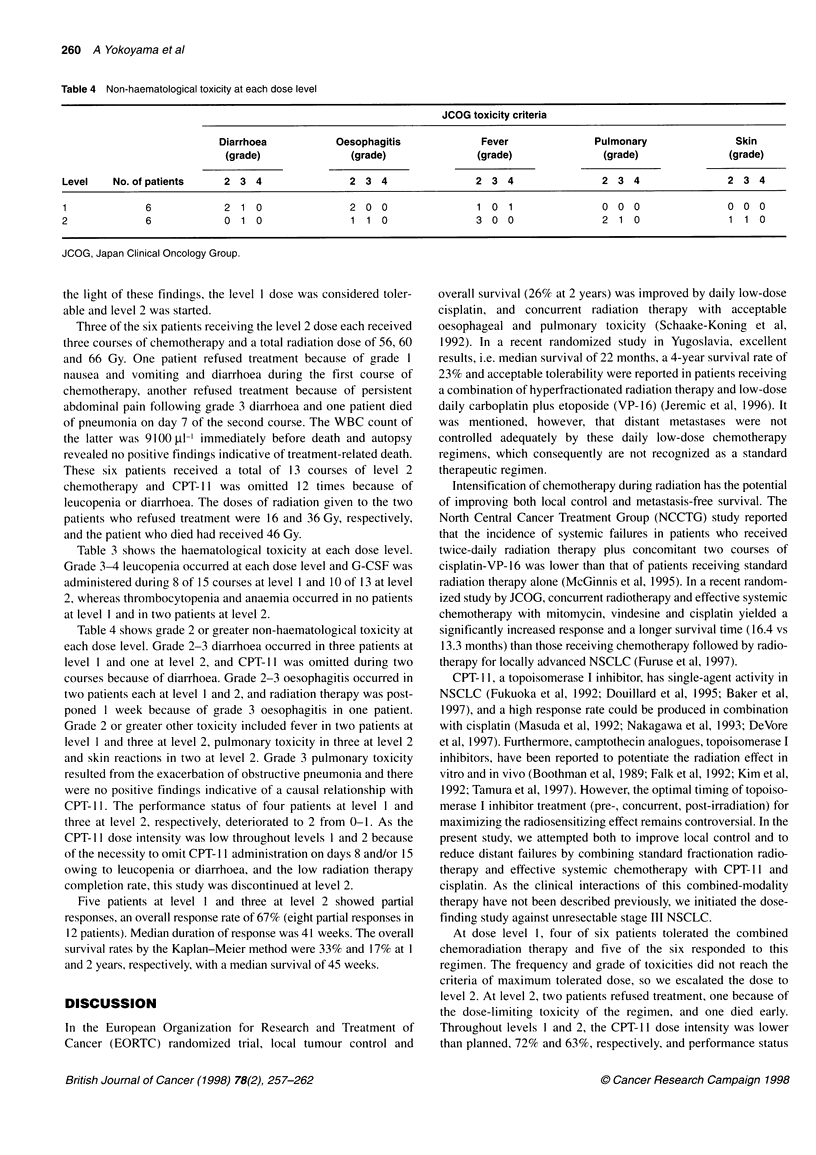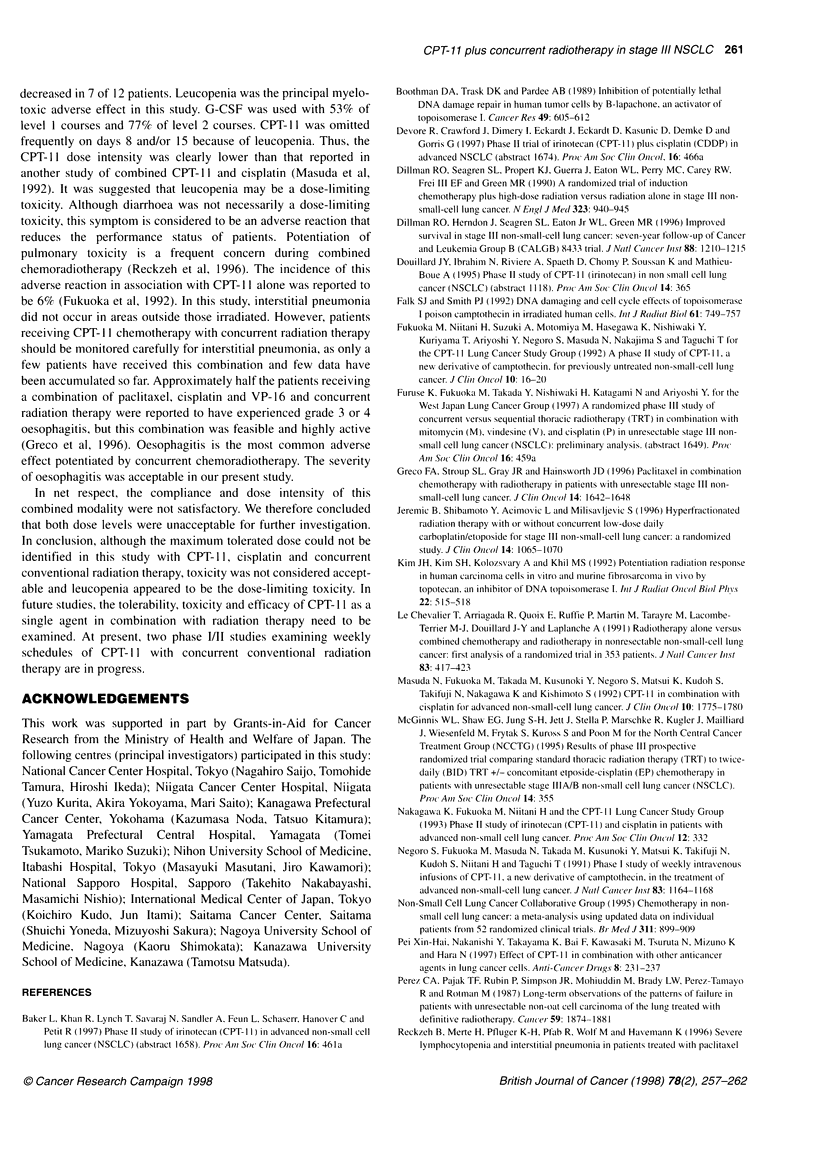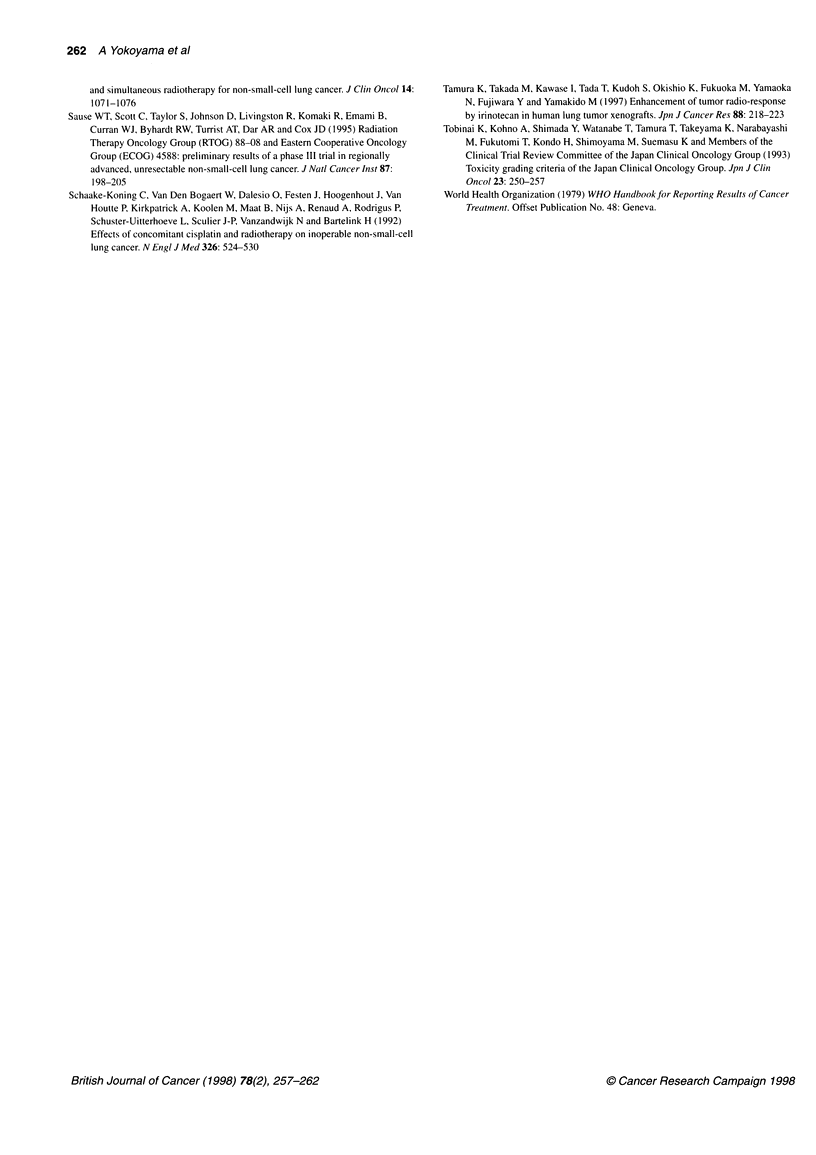# Dose-finding study of irinotecan and cisplatin plus concurrent radiotherapy for unresectable stage III non-small-cell lung cancer [seecomments]

**DOI:** 10.1038/bjc.1998.474

**Published:** 1998-07

**Authors:** A. Yokoyama, Y. Kurita, N. Saijo, T. Tamura, K. Noda, K. Shimokata, T. Matsuda

**Affiliations:** Department of Internal Medicine, Niigata Cancer Center Hospital, Japan.

## Abstract

Irinotecan hydrochloride (CPT-11) shows marked anti-tumour activity alone and in combination with cisplatin in non-small-cell lung cancer (NSCLC). It is necessary to investigate combined-modality therapy including novel effective anti-cancer agents to improve long-term survival of patients with unresectable stage III NSCLC. A phase I/II study of concurrent chemoradiotherapy with CPT-11 and cisplatin was conducted to determine the maximum tolerated dose (MTD) and efficacy in this group of patients. Thirteen previously untreated patients with unresectable stage IIIA/B NSCLC were enrolled and efficacy and toxicity was evaluated in 12 of them; one patient was ineligible. Chemotherapy was repeated every 4 weeks for three courses. Radiation therapy was started on day 2 of the first course of chemotherapy and 60 Gy in 30 fractions was given over 6 weeks. Four of six patients enrolled at level 1 completed the scheduled treatment. Another two received only one and two courses of chemotherapy as a result of persistent leucopenia and neutropenic fever respectively. Three of six patients given level 2 therapy completed the scheduled treatment. Another three received only one and two courses of chemotherapy, two refused treatment because of diarrhoea and one died of pneumonia. Radiation therapy was inadequate in these three patients. As the CPT-11 dose intensity in this trial was low, because of the necessity of omitting CPT-11 administration on days 8 and/or 15 as a result of leucopenia or diarrhoea, and the low radiation therapy completion rate, the trial was discontinued at level 2. Five patients at level 1 and three at level 2 showed partial responses, an overall response rate of 67%. Although neither MTD nor dose-limiting toxicity could be identified, chemotherapy with CPT-11 and cisplatin plus concurrent radiation therapy was deemed unacceptable. We are now conducting a phase I/II study of chemotherapy using CPT-11 as a single agent in combination with radiation therapy.


British Journal of Cancer (1998) 78(2), 257–262
© 1998 Cancer Research Campaign

### Dose-finding study of irinotecan and cisplatin plus concurrent radiotherapy for unresectable stage III non-small-cell lung cancer

A Yokoyama[1], Y Kurita[1], N Saijo[2], T Tamura[3], K Noda[4], K Shimokata[5] and T Matsuda[6]

[1]Department of Internal Medicine, Niigata Cancer Center Hospital, 2-15-3, Kawagishi-cho, Niigata, 951 Japan; [2]Pharmacology Division, National Cancer Center Research Institute, 1-1, Tsukiji 5-chome, Chuo-ku Tokyo, 104 Japan; [3]Department of Internal Medicine, National Cancer Center Hospital, 1-1, Tsukiji 5-chome, Chuo-ku Tokyo, 104 Japan; [4]Department of Internal Medicine, Kanagawa Prefectural Cancer Center, 54-2, Nakao-cho, Asahi-ku, Yokohama, 241 Japan; [5]First Department of Internal Medicine, Nagoya University School of Medicine, 65, Tsurumai-cho, Showa-ku, Nagoya, 466 Japan; [6]Third Department of Internal Medicine, Kanazawa University School of Medicine, 13-1, Takara-machi, Kanazawa, 920 Japan

**Summary** Irinotecan hydrochloride (CPT-11) shows marked anti-tumour activity alone and in combination with cisplatin in non-small-cell lung cancer (NSCLC). It is necessary to investigate combined-modality therapy including novel effective anti-cancer agents to improve long-term survival of patients with unresectable stage III NSCLC. A phase I/II study of concurrent chemoradiotherapy with CPT-11 and cisplatin was conducted to determine the maximum tolerated dose (MTD) and efficacy in this group of patients. Thirteen previously untreated patients with unresectable stage IIIA/B NSCLC were enrolled and efficacy and toxicity was evaluated in 12 of them; one patient was ineligible. Chemotherapy was repeated every 4 weeks for three courses. Radiation therapy was started on day 2 of the first course of chemotherapy and 60 Gy in 30 fractions was given over 6 weeks. Four of six patients enrolled at level 1 completed the scheduled treatment. Another two received only one and two courses of chemotherapy as a result of persistent leucopenia and neutropenic fever respectively. Three of six patients given level 2 therapy completed the scheduled treatment. Another three received only one and two courses of chemotherapy, two refused treatment because of diarrhoea and one died of pneumonia. Radiation therapy was inadequate in these three patients. As the CPT-11 dose intensity in this trial was low, because of the necessity of omitting CPT-11 administration on days 8 and/or 15 as a result of leucopenia or diarrhoea, and the low radiation therapy completion rate, the trial was discontinued at level 2. Five patients at level 1 and three at level 2 showed partial responses, an overall response rate of 67%. Although neither MTD nor dose-limiting toxicity could be identified, chemotherapy with CPT-11 and cisplatin plus concurrent radiation therapy was deemed unacceptable. We are now conducting a phase I/II study of chemotherapy using CPT-11 as a single agent in combination with radiation therapy.

**Keywords:** non-small-cell lung cancer; irinotecan; radiation therapy; combined-modality therapy

Approximately 25–30% of non-small cell lung cancer (NSCLC) is already unresectable stage III disease by the time it is diagnosed and, therefore, about 7000 such patients are seen in Japan per year. Traditionally, the standard treatment is radiation therapy, but the 5-year survival rate is 10% or less in patients treated with radiation therapy alone and the majority of patients experience extrathoracic recurrence (Perez et al, 1987). It is important to control distant metastases, as well as local tumours, in order to improve long-term survival. The efficacy of chemotherapy in such patients has long been a controversial issue. Recently, several randomized trials (Dillman et al, 1990, 1996; Le Chevalier et al, 1991; Sause et al, 1995) and meta-analysis (Non-small Cell Lung Cancer Collaborative Group, 1995) showed that chemotherapy followed by radiation therapy was more effective than radiation therapy alone. However, survival was still only prolonged by several months and it is necessary to investigate combined-modality therapy, including novel effective anti-cancer agents, and to establish the optimal timing and fractionation of radiation therapy.

*Received 26 March 1997*
*Revised 1 December 1997*
*Accepted 3 February 1998*

*Correspondence to*: A Yokoyama

Irinotecan hydrochloride (CPT-11), a new derivative of camptothecin, is one of the most active drugs against NSCLC (Fukuoka et al, 1992; Douillard et al, 1995; Baker et al, 1997). Furthermore, the addition of cisplatin to CPT-11 has been reported to result in synergistic cytotoxicity in preclinical models (Pei et al, 1997). Phase I/II studies of CPT-11 combined with cisplatin in patients with advanced NSCLC demonstrated a high response rate (31–48%) and a promising median survival (44 weeks) (Masuda et al, 1992; Nakagawa et al, 1993; DeVore et al, 1997). The administration of concomitant chemoradiotherapy has been shown to increase local and regional control of locally advanced NSCLC (Schaake-Koning et al, 1992). We considered that combined CPT-11 and cisplatin with concurrent radiation therapy could produce a high response rate and better survival outcome if it could be delivered at full dose. To our knowledge, no clinical studies on the concurrent use of CPT-11 and radiation therapy have been performed. Based on this idea the Japan Clinical Oncology Group (JCOG) initiated a dose-finding study of this combined modality. This study consisted of two phases: the first involved examining the optimal doses of CPT-11 and cisplatin plus concurrent radiation therapy by administering escalating doses of CPT-11 and cisplatin; and in the second, the anti-tumour effects and safety of this regimen were to be investigated using the recommended doses of CPT-11 and cisplating established in phase I.

### MATERIALS AND METHODS

### Patient selection

Patients with previously untreated NSCLC, diagnosed definitively histologically or cytologically, participated in this study. Patients were considered eligible if: they had unresectable stage IIIA or IIIB disease that was measurable, radical radiation therapy with a specified radiation field was possible, they were ≤ 75 years and had an Eastern Cooperative Oncology Group (ECOG) performance status of 0 or 1.

Before initiating treatment, a full medical history was obtained, physical examination was performed and definitive disease staging was carried out based on the following: complete blood cell count, serum chemistry, serum electrolytes, creatinine clearance, urinalysis, tumour markers, pulmonary function test, electrocardiogram (ECG), blood gas analysis, chest radiology, computerized tomography (chest, brain, abdomen), bronchoscopy and bone scan. All patients enrolled were required to have adequate organ function, i.e. WBC ≥ 4000 $\mu l^{-1}$, haemoglobin ≥ 10 g $dl^{-1}$, platelet count ≥ 10 × 104 $\mu l^{-1}$, total bilirubin ≤ 1.5 mg $dl^{-1}$, GOT/GPT ≤ normal value × 2, creatinine ≤ 1.5 mg $dl^{-1}$, creatinine clearance ≥ 60 ml $min^{-1}$ and $po_2$ ≥ 70 T. Patients with pleural effusion, apparent pericardial effusion, a history of malignancy within 5 years of entering the study, serious heart disease, uncontrollable diabetes, uncontrollable hypertension, chronic pulmonary disease that would make radiation therapy difficult and/or serious concomitant infection were excluded. All patients gave written informed consent before enrolment. The study was approved by the Clinical Trial Review Committee of JCOG and the ethics committee of each collaborating centre.

### Treatment plan

Eligible patients started treatment within 1 week of enrolment. Three courses of chemotherapy (CPT-11, days 1, 8 and 15; cisplatin, day 1) were repeated at 4-week intervals and concurrent radiation therapy was given once daily (2 Gy, 5 days per week from day 2 of the first course of chemotherapy). The total dose was intended to be 60 Gy in 30 fractions over a period of 6 weeks.

### Chemotherapy

CPT-11 was dissolved in 250 ml of 5% w/v glucose and administered i.v. over 90 min followed 2 h later by cisplatin, which was administered i.v. over 60 min on day 1. The participating researchers at each institution were allowed to decide how much fluid replacement and what antiemetic therapy should be administered, but adequate amounts of parenteral fluid and diuretics were to be given in order to prevent the renal toxicity of cisplatin.

### Dose escalation schedule

Dose escalation was planned in three levels, i.e. 40 mg $m^{-2}$ CPT-11 and 60 mg $m^{-2}$ cisplatin at level 1, 60 mg $m^{-2}$ CPT-11 and 60 mg $m^{-2}$ cisplatin at level 2 and 60 mg $m^{-2}$ CPT-11 and 80 mg $m^{-2}$ cisplatin at level 3. The initial dose of cisplatin was 60 mg $m^{-2}$, 75% of its recommended dose in combination chemotherapy. CPT-11 was initially given at a dose of 40 mg /$m^2$, 40% of its recommended dose as single agent (Negoro et al, 1991) and 66% of that recommended in combination with cisplatin (Masuda et al, 1992).

Six patients were enrolled at each level and evaluated on completion of the first course of chemotherapy and radiation therapy. If three of six patients had to discontinue treatment as a result of toxicity, dose escalation was to be discontinued, but if only two of six patients discontinued, it was to be continued to the next level. The doses of CPT-11 and cisplatin to be used for the phase II study were to be determined by examining carefully the toxicities resulting from second and third courses in particular, as well as the first, and the dose intensities of the two agents.

### Radiation therapy

A3- to 10-MeV linear accelerator with two posterioanterior opposed beams was used. The area of the lung field included in the radiation field was to be no greater than half the area of the unilateral lung. The radiation field was established 1.5 cm beyond the margin of the primary lesion and included the ispilateral hilar and mediastinal nodes. If metastasis to the supraclavicular node was found, this node was also to be included in the radiation field. Reduction of the radiation field was allowed, providing that at least 40 Gy had been administered and the researchers at each institution were allowed to use any suitable method to protect the spinal cord.

### Dose modification

### *Haematological toxicity*

CPT-11 was omitted if the WBC count was < 3000$\mu l^{-1}$ and/or platelet count was 75 × $10^3/\mu l^{-1}$ on day 8 (or day 15) of chemotherapy. If the WBC count was < 2000 $\mu l^{-1}$ or neutrophil count was < 1000 $\mu l^{-1}$ granulocyte colony-stimulating factor (G-CSF) was administered subcutaneously until recovery. The next course of chemotherapy was started after confirmation of WBC recovery to ≥ 3000 $\mu l^{-1}$ and platelets to ≥ 75 × $10^3$ $\mu l^{-1}$. If no recovery occurred, even after postponing the start of chemotherapy for ≥ 3 weeks, chemotherapy was to be discontinued. If grade 4 leucopenia or neutropenia continued for ≥ 4 days or grade 4 thrombocytopenia occurred during the previous chemotherapy course, the CPT-11 dose was reduced to 75% of that

**Table 1** Characteristics of the 12 eligible patients

| | |
|---|---|
| Age (years) | |
| Median | 58 |
| Range | 35–68 |
| Sex | |
| Male | 9 |
| Female | 3 |
| Performance status[a] | |
| 0 | 3 |
| 1 | 9 |
| Cell type | |
| Adenocarcinoma | 7 |
| Squamous | 4 |
| Large cell | 1 |
| Clinical stage | |
| IIIA | 2 |
| IIIB | 10 |

[a]According to the Eastern Cooperative Oncology Group.

© Cancer Research Campaign 1998

**Table 2** Dose escalation scheme and treatment given to patients at each dose level

| Level | Dose (mg m²) CPT-11 day 1, 8, 15 | Dose (mg m²) Cisplatin day 1 | No. of patients | No. of courses | No. of omitted[a] | % of ADDI[b] | TRT dose ≥ 60 Gy |
|---|---|---|---|---|---|---|---|
| 1 | 40 | 60 | 6 | 15 | 11 | 72 | 6 |
| 2 | 60 | 60 | 6 | 13 | 12 | 63 | 2 |
| 3 | 60 | 80 | – | | | | |

[a]No. of times that necessitated omitting CPT-11 administration on day 8 and/or day 15 because of leucopenia or diarrhoea. [b]Per cent of actually delivered dose intensity of CPT-11.

**Table 3** Haematological toxicity at each dose level

| Level | No. of patients/ no. of courses | JCOG toxicity criteria: WBC grade 3 | WBC grade 4 | Platelet grade 3 | Anaemia grade 3 | G-CSF No. of courses |
|---|---|---|---|---|---|---|
| 1 | 6/15 | 6/9 | 0/0 | 0/0 | 0/0 | 8 |
| 2 | 6/13 | 1/5 | 2/2 | 2/2 | 2/2 | 10 |

JCOG, Japan Clinical Oncology Group; G-CSF, granulocyte colony-stimulating factor.

specified for the next treatment course. If grade 4 haematological toxicity occurred during radiation therapy, radiation therapy was interrupted and restarted after recovery to grade 3 or less.

### *Diarrhoea*

If grade 2 or greater diarrhoea and/or abdominal pain occurred, CPT-11 was interrupted, and the next course was only started if the diarrhoea had resolved. If grade 3 or greater diarrhoea and/or abdominal pain had occurred during the previous chemotherapy course, the next CPT-11 dose was reduced to 75% of that specified.

### *Oesophagitis*

If grade 3 or greater oesophagitis occurred, radiation therapy was interrupted and restarted after recovery to grade 2 or less. If the severity of oesophagitis did not decrease to grade 2 or less even after 2 or more weeks, radiation therapy was discontinued.

### *Pulmonary toxicity*

If $po_2$ fell to 10 torr or lower, both radiation therapy and chemotherapy were interrupted and restarted as soon as possible after recovery. If diffuse interstitial pneumonia occurred, treatment was to be discontinued and steroid therapy instituted.

### *Fever*

If a patient had a fever of 38°C or higher, chemotherapy and radiation therapy were postponed until the fever subsided.

### *Other toxicities (excluding nausea/vomiting and alopecia)*

If other grade 2 or greater non-haematological toxicity occurred, treatment was postponed until recovery. If it was considered difficult to continue treatment even though toxicity was grade 2 or less, treatment was postponed until recovery. If treatment could not be restarted even after 2 or more weeks, treatment was discontinued.

Treatment duration was intended to be within 16 weeks for chemotherapy and within 12 weeks for radiation therapy. If treatment was not completed within these time periods, it was discontinued. Supportive therapies, e.g. loperamide to control late-onset diarrhoea, antibiotic therapy and transfusion, were to be given to control symptoms caused by treatment toxicity to the maximum possible extent.

### Response and toxicity evaluation

Responses were evaluated according to the World Health Organization (WHO) criteria (World Health Organization, 1979), and toxicity was assessed according to JCOG Toxicity Criteria (Tobinai et al, 1993). Since 1992, JCOG Toxicity Criteria have been used in all clinical trials conducted by JCOG. Most detailed gradings for individual organ toxicity in JCOG Toxicity Criteria are identical to those of WHO Toxicity Criteria (World Health Organization, 1979). All reported responses and toxicities were confirmed by independent extramural review.

### RESULTS

A total of 13 patients was enrolled between September 1994 and January 1995 and efficacy and toxicity could be evaluated in 12 of them. The remaining patient was ineligible because distant metastasis was confirmed after enrolment. Table 1 shows characteristics of the 12 eligible patients.

Table 2 shows treatment given to patients at each dose level. Four of the six patients at level 1 completed the scheduled three courses of chemotherapy and 60 Gy radiation therapy. One patient discontinued chemotherapy during the second course because of grade 3 leucopenia and grade 4 fever accompanied by hypotension and another did so during the first course because of persistent leucopenia. A total of 15 courses of chemotherapy was administered to the six patients, and CPT-11 was omitted 11 times because of leucopenia. Radiation therapy of 60 Gy was possible for all these patients, including those who discontinued chemotherapy. In

© Cancer Research Campaign 1998 

**Table 4** Non-haematological toxicity at each dose level

| | | JCOG toxicity criteria | | | | | | | | | | | | | | |
|---|---|---|---|---|---|---|---|---|---|---|---|---|---|---|---|---|
| | | Diarrhoea (grade) | | | Oesophagitis (grade) | | | Fever (grade) | | | Pulmonary (grade) | | | Skin (grade) | | |
| Level | No. of patients | 2 | 3 | 4 | 2 | 3 | 4 | 2 | 3 | 4 | 2 | 3 | 4 | 2 | 3 | 4 |
| 1 | 6 | 2 | 1 | 0 | 2 | 0 | 0 | 1 | 0 | 1 | 0 | 0 | 0 | 0 | 0 | 0 |
| 2 | 6 | 0 | 1 | 0 | 1 | 1 | 0 | 3 | 0 | 0 | 2 | 1 | 0 | 1 | 1 | 0 |

JCOG, Japan Clinical Oncology Group.

the light of these findings, the level 1 dose was considered tolerable and level 2 was started.

Three of the six patients receiving the level 2 dose each received three courses of chemotherapy and a total radiation dose of 56, 60 and 66 Gy. One patient refused treatment because of grade 1 nausea and vomiting and diarrhoea during the first course of chemotherapy, another refused treatment because of persistent abdominal pain following grade 3 diarrhoea and one patient died of pneumonia on day 7 of the second course. The WBC count of the latter was 9100 $\mu l^{-1}$ immediately before death and autopsy revealed no positive findings indicative of treatment-related death. These six patients received a total of 13 courses of level 2 chemotherapy and CPT-11 was omitted 12 times because of leucopenia or diarrhoea. The doses of radiation given to the two patients who refused treatment were 16 and 36 Gy, respectively, and the patient who died had received 46 Gy.

Table 3 shows the haematological toxicity at each dose level. Grade 3–4 leucopenia occurred at each dose level and G-CSF was administered during 8 of 15 courses at level 1 and 10 of 13 at level 2, whereas thrombocytopenia and anaemia occurred in no patients at level 1 and in two patients at level 2.

Table 4 shows grade 2 or greater non-haematological toxicity at each dose level. Grade 2–3 diarrhoea occurred in three patients at level 1 and one at level 2, and CPT-11 was omitted during two courses because of diarrhoea. Grade 2–3 oesophagitis occurred in two patients each at level 1 and 2, and radiation therapy was postponed 1 week because of grade 3 oesophagitis in one patient. Grade 2 or greater other toxicity included fever in two patients at level 1 and three at level 2, pulmonary toxicity in three at level 2 and skin reactions in two at level 2. Grade 3 pulmonary toxicity resulted from the exacerbation of obstructive pneumonia and there were no positive findings indicative of a causal relationship with CPT-11. The performance status of four patients at level 1 and three at level 2, respectively, deteriorated to 2 from 0–1. As the CPT-11 dose intensity was low throughout levels 1 and 2 because of the necessity to omit CPT-11 administration on days 8 and/or 15 owing to leucopenia or diarrhoea, and the low radiation therapy completion rate, this study was discontinued at level 2.

Five patients at level 1 and three at level 2 showed partial responses, an overall response rate of 67% (eight partial responses in 12 patients). Median duration of response was 41 weeks. The overall survival rates by the Kaplan–Meier method were 33% and 17% at 1 and 2 years, respectively, with a median survival of 45 weeks.

### DISCUSSION

In the European Organization for Research and Treatment of Cancer (EORTC) randomized trial, local tumour control and overall survival (26% at 2 years) was improved by daily low-dose cisplatin, and concurrent radiation therapy with acceptable oesophageal and pulmonary toxicity (Schaake-Koning et al, 1992). In a recent randomized study in Yugoslavia, excellent results, i.e. median survival of 22 months, a 4-year survival rate of 23% and acceptable tolerability were reported in patients receiving a combination of hyperfractionated radiation therapy and low-dose daily carboplatin plus etoposide (VP-16) (Jeremic et al, 1996). It was mentioned, however, that distant metastases were not controlled adequately by these daily low-dose chemotherapy regimens, which consequently are not recognized as a standard therapeutic regimen.

Intensification of chemotherapy during radiation has the potential of improving both local control and metastasis-free survival. The North Central Cancer Treatment Group (NCCTG) study reported that the incidence of systemic failures in patients who received twice-daily radiation therapy plus concomitant two courses of cisplatin-VP-16 was lower than that of patients receiving standard radiation therapy alone (McGinnis et al, 1995). In a recent randomized study by JCOG, concurrent radiotherapy and effective systemic chemotherapy with mitomycin, vindesine and cisplatin yielded a significantly increased response and a longer survival time (16.4 vs 13.3 months) than those receiving chemotherapy followed by radiotherapy for locally advanced NSCLC (Furuse et al, 1997).

CPT-11, a topoisomerase I inhibitor, has single-agent activity in NSCLC (Fukuoka et al, 1992; Douillard et al, 1995; Baker et al, 1997), and a high response rate could be produced in combination with cisplatin (Masuda et al, 1992; Nakagawa et al, 1993; DeVore et al, 1997). Furthermore, camptothecin analogues, topoisomerase I inhibitors, have been reported to potentiate the radiation effect in vitro and in vivo (Boothman et al, 1989; Falk et al, 1992; Kim et al, 1992; Tamura et al, 1997). However, the optimal timing of topoisomerase I inhibitor treatment (pre-, concurrent, post-irradiation) for maximizing the radiosensitizing effect remains controversial. In the present study, we attempted both to improve local control and to reduce distant failures by combining standard fractionation radiotherapy and effective systemic chemotherapy with CPT-11 and cisplatin. As the clinical interactions of this combined-modality therapy have not been described previously, we initiated the dose-finding study against unresectable stage III NSCLC.

At dose level 1, four of six patients tolerated the combined chemoradiation therapy and five of the six responded to this regimen. The frequency and grade of toxicities did not reach the criteria of maximum tolerated dose, so we escalated the dose to level 2. At level 2, two patients refused treatment, one because of the dose-limiting toxicity of the regimen, and one died early. Throughout levels 1 and 2, the CPT-11 dose intensity was lower than planned, 72% and 63%, respectively, and performance status

© Cancer Research Campaign 1998

decreased in 7 of 12 patients. Leucopenia was the principal myelotoxic adverse effect in this study. G-CSF was used with 53% of level 1 courses and 77% of level 2 courses. CPT-11 was omitted frequently on days 8 and/or 15 because of leucopenia. Thus, the CPT-11 dose intensity was clearly lower than that reported in another study of combined CPT-11 and cisplatin (Masuda et al, 1992). It was suggested that leucopenia may be a dose-limiting toxicity. Although diarrhoea was not necessarily a dose-limiting toxicity, this symptom is considered to be an adverse reaction that reduces the performance status of patients. Potentiation of pulmonary toxicity is a frequent concern during combined chemoradiotherapy (Reckzeh et al, 1996). The incidence of this adverse reaction in association with CPT-11 alone was reported to be 6% (Fukuoka et al, 1992). In this study, interstitial pneumonia did not occur in areas outside those irradiated. However, patients receiving CPT-11 chemotherapy with concurrent radiation therapy should be monitored carefully for interstitial pneumonia, as only a few patients have received this combination and few data have been accumulated so far. Approximately half the patients receiving a combination of paclitaxel, cisplatin and VP-16 and concurrent radiation therapy were reported to have experienced grade 3 or 4 oesophagitis, but this combination was feasible and highly active (Greco et al, 1996). Oesophagitis is the most common adverse effect potentiated by concurrent chemoradiotherapy. The severity of oesophagitis was acceptable in our present study.

In net respect, the compliance and dose intensity of this combined modality were not satisfactory. We therefore concluded that both dose levels were unacceptable for further investigation. In conclusion, although the maximum tolerated dose could not be identified in this study with CPT-11, cisplatin and concurrent conventional radiation therapy, toxicity was not considered acceptable and leucopenia appeared to be the dose-limiting toxicity. In future studies, the tolerability, toxicity and efficacy of CPT-11 as a single agent in combination with radiation therapy need to be examined. At present, two phase I/II studies examining weekly schedules of CPT-11 with concurrent conventional radiation therapy are in progress.

### ACKNOWLEDGEMENTS

This work was supported in part by Grants-in-Aid for Cancer Research from the Ministry of Health and Welfare of Japan. The following centres (principal investigators) participated in this study: National Cancer Center Hospital, Tokyo (Nagahiro Saijo, Tomohide Tamura, Hiroshi Ikeda); Niigata Cancer Center Hospital, Niigata (Yuzo Kurita, Akira Yokoyama, Mari Saito); Kanagawa Prefectural Cancer Center, Yokohama (Kazumasa Noda, Tatsuo Kitamura); Yamagata Prefectural Central Hospital, Yamagata (Tomei Tsukamoto, Mariko Suzuki); Nihon University School of Medicine, Itabashi Hospital, Tokyo (Masayuki Masutani, Jiro Kawamori); National Sapporo Hospital, Sapporo (Takehito Nakabayashi, Masamichi Nishio); International Medical Center of Japan, Tokyo (Koichiro Kudo, Jun Itami); Saitama Cancer Center, Saitama (Shuichi Yoneda, Mizuyoshi Sakura); Nagoya University School of Medicine, Nagoya (Kaoru Shimokata); Kanazawa University School of Medicine, Kanazawa (Tamotsu Matsuda).

### REFERENCES

Baker L, Khan R, Lynch T, Savaraj N, Sandler A, Feun L, Schaserr, Hanover C and Petit R (1997) Phase II study of irinotecan (CPT-11) in advanced non-small cell lung cancer (NSCLC) (abstract 1658). *Proc Am Soc Clin Oncol* **16**: 461a

Boothman DA, Trask DK and Pardee AB (1989) Inhibition of potentially lethal DNA damage repair in human tumor cells by B-lapachone, an activator of topoisomerase I. *Cancer Res* **49**: 605–612

Devore R, Crawford J, Dimery I, Eckardt J, Eckardt D, Kasunic D, Demke D and Gorris G (1997) Phase II trial of irinotecan (CPT-11) plus cisplatin (CDDP) in advanced NSCLC (abstract 1674). *Proc Am Soc Clin Oncol*, **16**: 466a

Dillman RO, Seagren SL, Propert KJ, Guerra J, Eaton WL, Perry MC, Carey RW, Frei III EF and Green MR (1990) A randomized trial of induction chemotherapy plus high-dose radiation versus radiation alone in stage III non-small-cell lung cancer. *N Engl J Med* **323**: 940–945

Dillman RO, Herndon J, Seagren SL, Eaton Jr WL, Green MR (1996) Improved survival in stage III non-small-cell lung cancer: seven-year follow-up of Cancer and Leukemia Group B (CALGB) 8433 trial. *J Natl Cancer Inst* **88**: 1210–1215

Douillard JY, Ibrahim N, Riviere A, Spaeth D, Chomy P, Soussan K and Mathieu-Boue A (1995) Phase II study of CPT-11 (irinotecan) in non small cell lung cancer (NSCLC) (abstract 1118). *Proc Am Soc Clin Oncol* **14**: 365

Falk SJ and Smith PJ (1992) DNA damaging and cell cycle effects of topoisomerase I poison camptothecin in irradiated human cells. *Int J Radiat Biol* **61**: 749–757

Fukuoka M, Niitani H, Suzuki A, Motomiya M, Hasegawa K, Nishiwaki Y, Kuriyama T, Ariyoshi Y, Negoro S, Masuda N, Nakajima S and Taguchi T for the CPT-11 Lung Cancer Study Group (1992) A phase II study of CPT-11, a new derivative of camptothecin, for previously untreated non-small-cell lung cancer. *J Clin Oncol* **10**: 16–20

Furuse K, Fukuoka M, Takada Y, Nishiwaki H, Katagami N and Ariyoshi Y, for the West Japan Lung Cancer Group (1997) A randomized phase III study of concurrent versus sequential thoracic radiotherapy (TRT) in combination with mitomycin (M), vindesine (V), and cisplatin (P) in unresectable stage III non-small cell lung cancer (NSCLC): preliminary analysis. (abstract 1649). *Proc Am Soc Clin Oncol* **16**: 459a

Greco FA, Stroup SL, Gray JR and Hainsworth JD (1996) Paclitaxel in combination chemotherapy with radiotherapy in patients with unresectable stage III non-small-cell lung cancer. *J Clin Oncol* **14**: 1642–1648

Jeremic B, Shibamoto Y, Acimovic L and Milisavljevic S (1996) Hyperfractionated radiation therapy with or without concurrent low-dose daily carboplatin/etoposide for stage III non-small-cell lung cancer: a randomized study. *J Clin Oncol* **14**: 1065–1070

Kim JH, Kim SH, Kolozsvary A and Khil MS (1992) Potentiation radiation response in human carcinoma cells in vitro and murine fibrosarcoma in vivo by topotecan, an inhibitor of DNA topoisomerase I. *Int J Radiat Oncol Biol Phys* **22**: 515–518

Le Chevalier T, Arriagada R, Quoix E, Ruffie P, Martin M, Tarayre M, Lacombe-Terrier M-J, Douillard J-Y and Laplanche A (1991) Radiotherapy alone versus combined chemotherapy and radiotherapy in nonresectable non-small-cell lung cancer: first analysis of a randomized trial in 353 patients. *J Natl Cancer Inst* **83**: 417–423

Masuda N, Fukuoka M, Takada M, Kusunoki Y, Negoro S, Matsui K, Kudoh S, Takifuji N, Nakagawa K and Kishimoto S (1992) CPT-11 in combination with cisplatin for advanced non-small-cell lung cancer. *J Clin Oncol* **10**: 1775–1780

McGinnis WL, Shaw EG, Jung S-H, Jett J, Stella P, Marschke R, Kugler J, Mailliard J, Wiesenfeld M, Frytak S, Kuross S and Poon M for the North Central Cancer Treatment Group (NCCTG) (1995) Results of phase III prospective randomized trial comparing standard thoracic radiation therapy (TRT) to twice-daily (BID) TRT +/– concomitant etposide-cisplatin (EP) chemotherapy in patients with unresectable stage IIIA/B non-small cell lung cancer (NSCLC). *Proc Am Soc Clin Oncol* **14**: 355

Nakagawa K, Fukuoka M, Niitani H and the CPT-11 Lung Cancer Study Group (1993) Phase II study of irinotecan (CPT-11) and cisplatin in patients with advanced non-small cell lung cancer. *Proc Am Soc Clin Oncol* **12**: 332

Negoro S, Fukuoka M, Masuda N, Takada M, Kusunoki Y, Matsui K, Takifuji N, Kudoh S, Niitani H and Taguchi T (1991) Phase I study of weekly intravenous infusions of CPT-11, a new derivative of camptothecin, in the treatment of advanced non-small-cell lung cancer. *J Natl Cancer Inst* **83**: 1164–1168

Non-Small Cell Lung Cancer Collaborative Group (1995) Chemotherapy in non-small cell lung cancer: a meta-analysis using updated data on individual patients from 52 randomized clinical trials. *Br Med J* **311**: 899–909

Pei Xin-Hai, Nakanishi Y, Takayama K, Bai F, Kawasaki M, Tsuruta N, Mizuno K and Hara N (1997) Effect of CPT-11 in combination with other anticancer agents in lung cancer cells. *Anti-Cancer Drugs* **8**: 231–237

Perez CA, Pajak TF, Rubin P, Simpson JR, Mohiuddin M, Brady LW, Perez-Tamayo R and Rotman M (1987) Long-term observations of the patterns of failure in patients with unresectable non-oat cell carcinoma of the lung treated with definitive radiotherapy. *Cancer* **59**: 1874–1881

Reckzeh B, Merte H, Pfluger K-H, Pfab R, Wolf M and Havemann K (1996) Severe lymphocytopenia and interstitial pneumonia in patients treated with paclitaxel

© Cancer Research Campaign 1998 

and simultaneous radiotherapy for non-small-cell lung cancer. *J Clin Oncol* **14**: 1071–1076

Sause WT, Scott C, Taylor S, Johnson D, Livingston R, Komaki R, Emami B, Curran WJ, Byhardt RW, Turrist AT, Dar AR and Cox JD (1995) Radiation Therapy Oncology Group (RTOG) 88–08 and Eastern Cooperative Oncology Group (ECOG) 4588: preliminary results of a phase III trial in regionally advanced, unresectable non-small-cell lung cancer. *J Natl Cancer Inst* **87**: 198–205

Schaake-Koning C, Van Den Bogaert W, Dalesio O, Festen J, Hoogenhout J, Van Houtte P, Kirkpatrick A, Koolen M, Maat B, Nijs A, Renaud A, Rodrigus P, Schuster-Uitterhoeve L, Sculier J-P, Vanzandwijk N and Bartelink H (1992) Effects of concomitant cisplatin and radiotherapy on inoperable non-small-cell lung cancer. *N Engl J Med* **326**: 524–530

Tamura K, Takada M, Kawase I, Tada T, Kudoh S, Okishio K, Fukuoka M, Yamaoka N, Fujiwara Y and Yamakido M (1997) Enhancement of tumor radio-response by irinotecan in human lung tumor xenografts. *Jpn J Cancer Res* **88**: 218–223

Tobinai K, Kohno A, Shimada Y, Watanabe T, Tamura T, Takeyama K, Narabayashi M, Fukutomi T, Kondo H, Shimoyama M, Suemasu K and Members of the Clinical Trial Review Committee of the Japan Clinical Oncology Group (1993) Toxicity grading criteria of the Japan Clinical Oncology Group. *Jpn J Clin Oncol* **23**: 250–257

World Health Organization (1979) *WHO Handbook for Reporting Results of Cancer Treatment*. Offset Publication No. 48: Geneva.

*© Cancer Research Campaign 1998*